# Single Cell RNAseq Analysis of Cytokine-Treated Human Islets: Association of Cellular Stress with Impaired Cytokine Responsiveness

**DOI:** 10.1093/function/zqae015

**Published:** 2024-03-27

**Authors:** Jennifer S Stancill, Moujtaba Y Kasmani, Weiguo Cui, John A Corbett

**Affiliations:** Department of Biochemistry and Molecular Biology, Medical University of South Carolina, Charleston, SC 29425, USA; Department of Microbiology and Immunology, Medical College of Wisconsin, Milwaukee, WI 53226, USA; Blood Research Institute, Versiti, Wisconsin, Milwaukee, WI 53226, USA; Department of Microbiology and Immunology, Medical College of Wisconsin, Milwaukee, WI 53226, USA; Blood Research Institute, Versiti, Wisconsin, Milwaukee, WI 53226, USA; Department of Biochemistry, Medical College of Wisconsin, Milwaukee, WI 53226, USA

**Keywords:** beta-cells, cytokines, inflammation, islets, pancreas, single-cell RNA-seq

## Abstract

Pancreatic β-cells are essential for survival, being the only cell type capable of insulin secretion. While they are believed to be vulnerable to damage by inflammatory cytokines such as interleukin-1 beta (IL-1β) and interferon-gamma, we have recently identified physiological roles for cytokine signaling in rodent β-cells that include the stimulation of antiviral and antimicrobial gene expression and the inhibition of viral replication. In this study, we examine cytokine-stimulated changes in gene expression in human islets using single-cell RNA sequencing. Surprisingly, the global responses of human islets to cytokine exposure were remarkably blunted compared to our previous observations in the mouse. The small population of human islet cells that were cytokine responsive exhibited increased expression of IL-1β-stimulated antiviral guanylate-binding proteins, just like in the mouse. Most human islet cells were not responsive to cytokines, and this lack of responsiveness was associated with high expression of genes encoding ribosomal proteins. We further correlated the expression levels of *RPL5* with stress response genes, and when expressed at high levels, *RPL5* is predictive of failure to respond to cytokines in all endocrine cells. We postulate that donor causes of death and isolation methodologies may contribute to stress of the islet preparation. Our findings indicate that activation of stress responses in human islets limits cytokine-stimulated gene expression, and we urge caution in the evaluation of studies that have examined cytokine-stimulated gene expression in human islets without evaluation of stress-related gene expression.

## Graphical Abstract

**Graphical Abstract:**
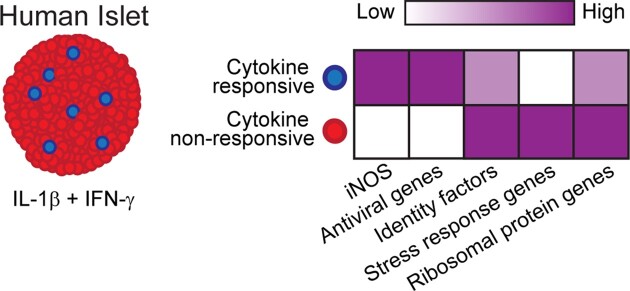

## Introduction

Pancreatic β-cells, responsible for synthesis and secretion of insulin in response to a glucose challenge, reside in the islets of Langerhans and are essential for survival of the organism as the only cell type capable of producing this hormone. In the absence of pancreatic β-cells, which occurs during autoimmune-mediated destruction, type 1 diabetes ensues. While the killing of these cells is primarily mediated by T-cell-dependent mechanisms,^1–3^ inflammatory cytokines like interleukin-1 beta (IL-1β) and interferon-gamma (IFN-γ) produced by macrophages and T-lymphocytes^4^,^5^ are believed to contribute to β-cell damage and disease development. Islets are highly vascularized and receive a disproportionately large amount of blood flow, a feature that is crucial for proper blood glucose control but also facilitates exposure of β-cells to circulating cytokines produced during a viral or bacterial infection.^6^,^7^ It has long been known that these cytokines stimulate β-cells to express inducible nitric oxide synthase (iNOS), and the resulting production of micromolar levels of nitric oxide inhibit mitochondrial oxidation (aconitase activity and electron transport) and insulin secretion. Nitric oxide also causes ER stress and activation of the unfolded protein response, induces DNA damage, and can cause cell death following prolonged exposure.^8–13^

Although these effects of cytokines have been viewed as damaging to β-cells, our recent studies suggest that there are physiological roles for cytokine signaling in the endocrine islet that are aimed at protecting the β-cells from environmental threats. Interleukin-1 beta stimulates expression of a subset of antiviral and antimicrobial genes in β-cells and other islet endocrine cell types in a nitric oxide-independent manner.^14^,^15^ Nitric oxide, by inhibiting mitochondrial oxidation, attenuates viral replication in a β-cell-selective manner, and nitric oxide is a potent inhibitor of insulinoma cell apoptosis.^16^,^17^ Importantly the inhibition of mitochondrial oxidation and insulin secretion are completely reversible, and β-cells have efficient mechanisms to repair damaged DNA; it is only prolonged incubations with IL-1β (greater than 36 h in vitro) that lead to irreversible damage.^18–21^ Together, these previous studies performed in rodent and human islets support a physiological model in which IL-1β signals to islet endocrine cells to increase the expression of protective, anti-pathogen factors.

Early studies identified a role of nitric oxide as a mediator of the inhibitory actions of cytokines on insulin secretion by human islets^12^,^22^; however, this effect has been challenged by studies that suggest that the response of human islets to cytokines differs from the response of rodent islets.^23–25^ For example, it has been suggested that, while cytokines stimulate nitric oxide production in human islets, the inhibition of insulin secretion by cytokines is independent of nitric oxide.^23^,^25^ In fact, most recent studies do not examine whether human or rodent islets produce nitric oxide in response to cytokines or if the observed responses are nitric oxide dependent.^26–28^ Observed differences between rodent and human islets are not limited to cytokine signaling.^29^ Of note, it has also been reported that human islets express higher levels of heat shock proteins than rodent islets.^30^,^31^ Because of these reported differences, it is critical to determine if IL-1β promotes protective responses in human β-cells.

In this study, we aimed to determine, by single-cell RNA sequencing (scRNA-seq), the effects of cytokines on gene expression in human islets. Specifically, we focused on identifying the early responses following a short 6-h exposure and nitric oxide-dependent responses following a longer 18-h exposure. This approach also allowed us to characterize the heterogeneity of human islet cell types with respect to their cytokine response. Over 45,000 cells were analyzed from 3 independent, non-diabetic human islet preparations exposed to IL-1β and IFN-γ with or without the nitric oxide synthase inhibitor N^G^-monomethyl-l-arginine (NMMA) for 6 or 18 h. In comparison to our previous analyses of mouse islet cells exposed to similar cytokine treatments,^14^,^15^ human β-cells demonstrated markedly blunted responses to cytokine stimulation, both in terms of the percentage of “responsive” cells and in terms of the percentage of differentially expressed genes. This blunted response was also observed in human α-, δ-, and PP-cells. We did identify a small subset of *NOS2*^+^ β-cells with similar cytokine-stimulated gene expression changes to those previously observed in the mouse, suggesting that the same signaling events likely occur in both species. The *NOS2*^−^ β-cells had higher expression of genes encoding several ribosomal proteins and heat shock proteins compared to the *NOS2*^+^ cells. One of these ribosomal protein-encoding genes, *RPL5*, was positively correlated with indicators of cellular stress and was negatively correlated with cytokine-stimulated genes. Importantly, high expression of *RPL5* predicted failure to respond to cytokines. This negative association between cytokine responsiveness and ribosomal protein expression was observed not only in β-cells but also in endocrine non-β-cells and non-endocrine cells. Our results dovetail with previous reports that cell stress, either by heat shock or induction of ER stress, inhibits cytokine signaling in rodent and human islets.^32–35^ Taken together, our results suggest that there are no major species differences in the response of islet cells to cytokines and that the major differences are in the induction of a stress response. Therefore, caution should be used when interpreting results of studies using isolated human islets that do not assess stress-related genes, as stress is a well-defined repressor of cytokine signaling and may impact multiple metabolic and signaling events.

## Materials and Methods

### Materials and Islets

**Figure 1.**
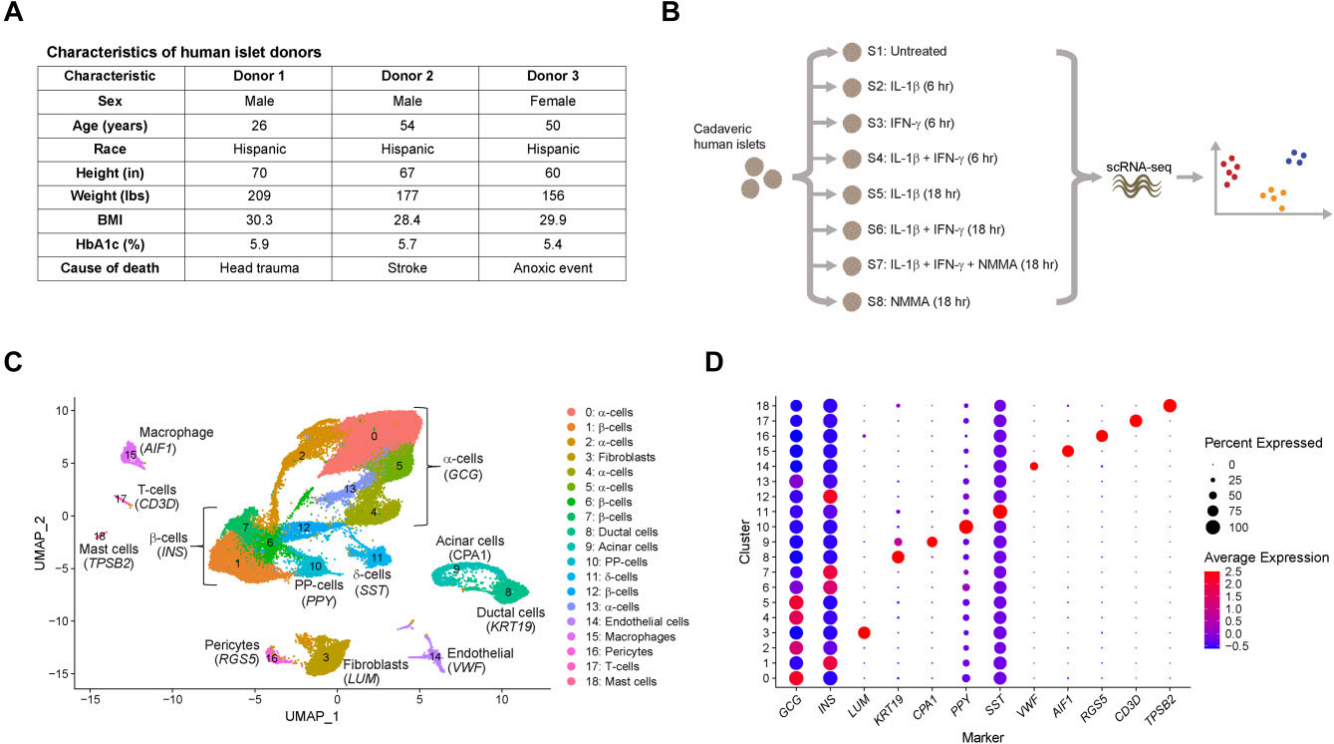
scRNA-sequencing of human islet following cytokine exposure. (A) Characteristics of human islet donors. (B) Schematic of experimental design. (C) Uniform manifold approximation and projection plot showing clusters of cells from all 3 scRNA-seq experimental replicates. Cell identity is shown to the right of the plot and was assigned based on enrichment for the genes indicated. (D) Dot plot indicating expression levels of and percentage of cells expressing marker genes in each of the 19 clusters.

Cadaveric human islets from 3 non-diabetic donors were obtained from Prodo Laboratories (Aliso Viejo, CA). Donor information is provided in Figure 1A. Connaught Medical Research Laboratories (CMRL) 1066 medium, Hank’s Balanced Salt Solution (HBSS), HEPES, sodium pyruvate, ι-glutamine, penicillin, and streptomycin were purchased from Thermo Fisher Scientific (Waltham, MA). Fetal bovine serum (FBS) is from HyClone (Logan, UT). Human recombinant IL-1β and IFN-γ were obtained from PeproTech (Rocky Hill, NJ). N^G^-Monomethyl-l-arginine is from Enzo Life Sciences (Farmingdale, NY).

### Islet Isolation, Culture, and Treatment

Pancreatic islets were cultured for 2–5 d after receipt at 37°C and 5% CO_2_ in CMRL supplemented with 10% heat-inactivated FBS and containing 5.5 mM glucose as previously described.^36^ Intact islets were left untreated or were treated with following cytokines: IL-1β, IFN-γ, or IL-1β + IFN-γ for 6 or 18 h. We also examined the effects of the NOS inhibitor NMMA on islets treated for 18 h with or without IL-1β + IFN-γ. Human recombinant IL-1β was used at a concentration of 50 U/mL, human recombinant IFN-γ at a concentration of 500 U/mL, and NMMA at a concentration of 2 mM. Independent scRNAseq experiments were performed using human islets from 3 different donors.

### Single-Cell RNA-Sequencing of Human Islets

Following treatment with cytokines, islets were incubated in 0.48 mM EDTA in phosphate-buffered saline and then agitated in 1 mg/mL trypsin in Ca^2+^/Mg^2+^-free HBSS to disperse into single cells. Cells were filtered and resuspended in CMRL media before being loaded into the Chromium Controller (10x Genomics). A wetting failure occurred during the generation of gel beads in emulsion (GEMs) of sample 5 (18 h IL-1β treatment) from the first experimental replicate. For this reason, this sample was excluded from further analyses. scRNA-seq libraries were prepared using the Chromium Single Cell 3′ v3 Reagent Kit (10x Genomics) for replicate 1 and the Chromium Next GEM Single Cell 3′ v3.1 Reagent Kit (10x Genomics) for replicates 2 and 3 according to the manufacturer’s protocol. Libraries were sequenced using the NextSeq 500/550 High Output Kit v2.5 flow cell (150 cycles, Illumina) according to the 10x Genomics protocol. Samples were sequenced to a depth that returned approximately 30,000 reads per cell on average. CellRanger (10x Genomics) functions “mkfastq” and “count” were used to demultiplex the sequencing data and generate gene-barcode matrices (10x Genomics). Reads were aligned to human reference genome assembly hg38. All scRNA-seq analysis was performed in R (version 4.3.1) using the package Seurat (version 4.0.0).^37^ Number of genes detected per cell and percent of mitochondrial genes were plotted, and outlier cells were removed [number of genes fewer than 200 or greater than 3500 (replicate 1) or 5500 (replicates 2 and 3) or percent mitochondrial genes over 10% (replicate 1) or 15% (replicates 2 and 3)] to filter out doublets and cells with low read quality, leaving 47,121 of the original 70,236 cells. Cell cycle genes were regressed. Seurat function “SCTransform” was used to integrate samples from all 3 experimental replicates into 1 dataset to reduce batch effects.^38^ Principal component analysis was performed, and the top 50 principal components were used for Uniform Manifold Approximation and Projection (UMAP) analysis, with clustering performed using the Louvain algorithm. All samples were normalized using Seurat’s default normalization settings.

### Mouse Islet Scrna-Seq Datasets

Our previously published single-cell RNA-sequencing datasets using mouse islets exposed to cytokines for 6 or 18 h were used for comparison to human islets. Data can be found under GEO accession numbers GSE156175 and GSE183010.^14^,^15^

### Functional Annotation Clustering Analysis

Lists of differentially expressed genes were inputted into the web interface of Database for Annotation, Visualization, and Integrated Discovery^39^ and were analyzed for enriched gene categories using the default settings. Similar annotations are grouped into “functional annotation clusters” and given an enrichment score defined as the geometric mean in −log scale of the *P*-values of the annotations in the functional annotation cluster. A higher enrichment score reflects lower *P*-values in the group and is more likely to be biologically meaningful.

### Statistical Analysis

For differential expression analyses, *P*-values were calculated using the Wilcoxon test, and Bonferroni correction was used to avoid false positives. An average log_2_ (fold change) of 0.25 and adjusted *P*-value of 0.05 were the threshold used to declare significance. Fisher’s exact test was used to compare percentages of genes changed by cytokine treatment in human islet cells to mouse islet cells. Linear regression analyses were performed using the ordinary least squares method in R using only cells with positive expression values of the genes being compared.

## Results

### Single-Cell RNA-Sequencing of Human Islets Following Cytokine Exposure

To understand the heterogenous effects of inflammatory cytokines on gene expression in the individual cell types found in human islets, we performed single-cell RNA-sequencing (scRNA-seq) using islets isolated from 3 non-diabetic cadaveric donors (Figure 1A) following treatment with inflammatory cytokines for either 6 or 18 h. Islets were untreated; treated for 6 h with IL-1β, IFN-γ, or IL-1β + IFN-γ; or treated for 18 h with IL-1β, IL-1β + IFN-γ, IL-1β + IFN-γ + NMMA, or NMMA alone (Figure 1B). Islets from each donor were treated with all conditions for a total of 3 independent scRNA-seq replicates. The combination of IL-1β + IFN-γ was chosen because this is the minimal cytokine combination necessary to stimulate iNOS expression and nitric oxide production in human islets.^12^,^40^ Cells from all samples and all 3 donors were combined into 1 dataset and were visualized using UMAP. After quality control, we were left with 47,121 cells that were grouped unbiasedly into 19 clusters based on similarity of gene expression (Figure 1C and Table S1). Cells from each donor contributed to every cluster, and cells from each treatment contributed to every cluster (Figure S1). We assigned endocrine cell identities (β-, α-, δ-, and PP-cells) based on enrichment of genes encoding the primary islet hormones (insulin, glucagon, somatostatin, and pancreatic polypeptide, respectively) (Figure 1D). α-cells (*GCG*) comprised 50% (23,354 cells) of our dataset, β-cells (*INS*) 23% (10,616 cells), PP-cells (*PPY*) 4% (1,681 cells), and δ-cells (*SST*) 3% (1,616 cells). Using characteristic gene expression, we also identified the cell types of the non-endocrine clusters, which made up the remaining 20% of our dataset: fibroblasts (*LUM*), ductal cells (*KRT19*), acinar cells (*CPA1*), endothelial cells (*VWF*), macrophages (*AIF1*), pericytes (*RGS5*), T-cells (*CD3D*), and mast cells (*TPSB2*) (Figure 1C and D).

### Human β-Cells Have a Blunted Cytokine Response Compared to Mouse β-Cells

**Figure 2.**
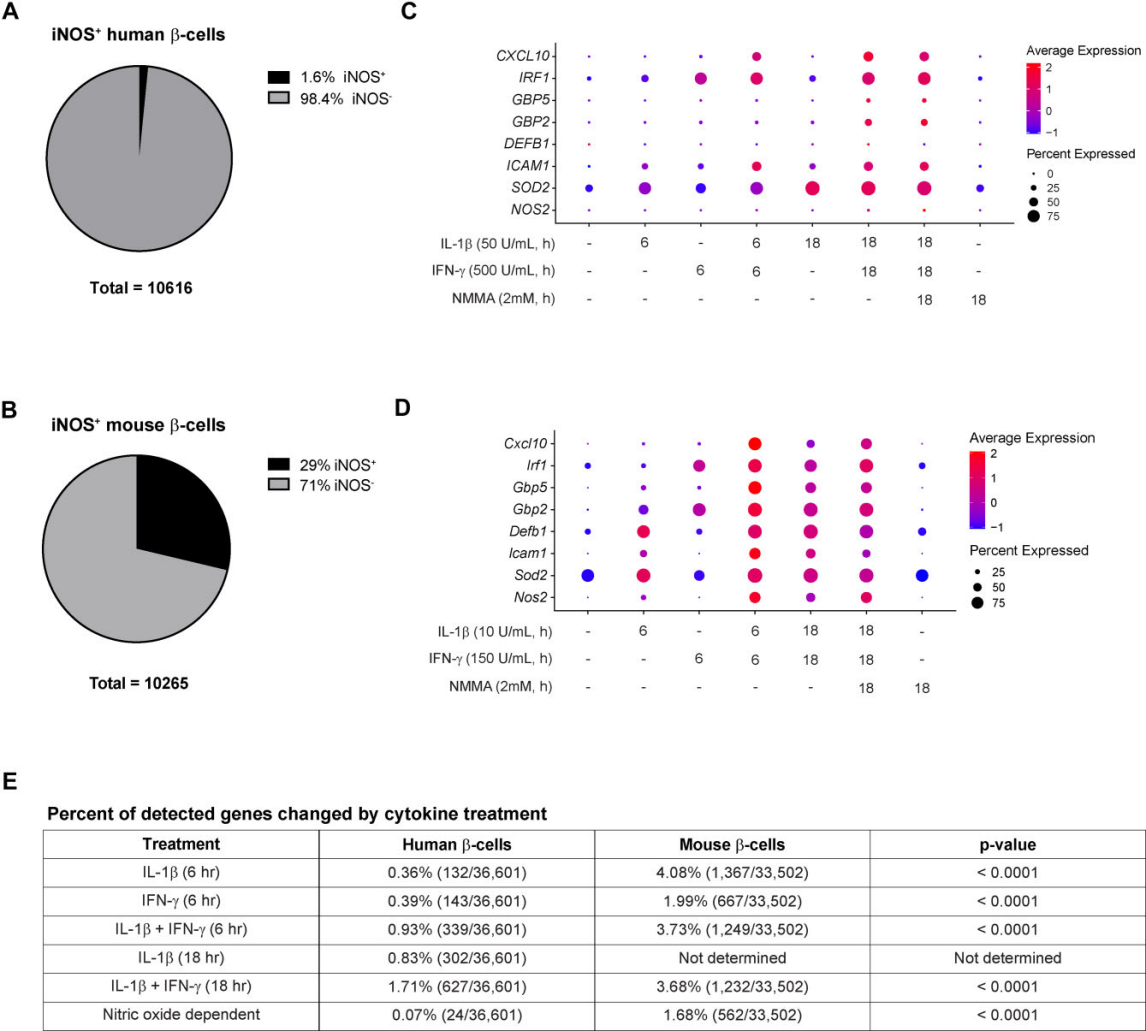
Human β-cells have a blunted cytokine response compared to mouse β-cells. (A and B) Pie chart showing the percentage of human (A) or mouse (B) β-cells from all samples that express iNOS mRNA. (C and D) Dot plots depicting the expression levels of and percentage of human (C) or mouse (D) β-cells expressing selected genes in response to each of the cytokine treatments. (E) Table summarizing the percent of detected genes that were significantly changed by each cytokine treatment or genes that were significantly changed in a nitric oxide-dependent manner in human and mouse β-cells. *P*-value determined by Fisher’s exact test. Mouse data are from our previously published studies.^14^,^15^

To determine how cytokine exposure alters human β-cell gene expression, we computationally isolated Clusters 1, 6, 7, and 12 from the total dataset (Figure 1C). Because stimulation of iNOS mRNA (*NOS2*) is a well-characterized response of β-cells to IL-1β + IFN-γ exposure,^12^ we used expression of this gene as a metric to assess the responsiveness of the human β-cell population to cytokines. Surprisingly, only 1.6% (168/10,616) of the total human β-cell population expressed detectable levels of *NOS2* (Figure 2A). This percentage is much lower than we observed in our previous single-cell RNA-sequencing studies using mouse islets.^14^,^15^ In those studies, 29% (2,948/10,265) of the whole β-cell population expressed *Nos2* (Figure 2B) despite similar cytokine exposure conditions *in vitro*. This blunted response of the human β-cells was not limited to *NOS2*. Visualization of the expression level and the percentage of cells expressing other selected cytokine-stimulated genes shows a markedly blunted response in human β-cells compared to mouse β-cells (Figure 2C and D).

As an unbiased approach to determine genes significantly changed in response to each cytokine treatment (compared to the untreated sample), we performed differential expression analysis of the human β-cell population (Table S2). Relative to untreated controls, following a 6-h treatment, a total of 132 genes (0.36% of the detected genes) were significantly changed in response to IL-1β, 143 (0.39%) in response to IFN-γ, and 339 (0.93%) in response IL-1β + IFN-γ (Figure 2E). Relative to untreated controls, following an 18-h treatment, a total of 302 (0.83%) genes were changed in response to IL-1β and 627 (1.71%) in response to IL-1β + IFN-γ (Figure 2E). By performing a similar differential expression analysis, but this time comparing genes changed in response to 18-h IL-1β + IFN-γ to those changed in response to 18-h IL-1β + IFN-γ + NMMA, we determined that the expression of only 24 genes (0.07% of the detected genes) was significantly changed by nitric oxide (Figure 2E). These numbers are significantly lower than we might expect based on the number of genes changed by each cytokine treatment in mouse β-cells (Figure 2E). Together, these observations suggest that human β-cells have a blunted gene expression response to cytokines compared to mouse β-cells.

### Cytokine Responsiveness is Also Blunted in Human Islet Endocrine Non-β-Cell Types

**Figure 3.**
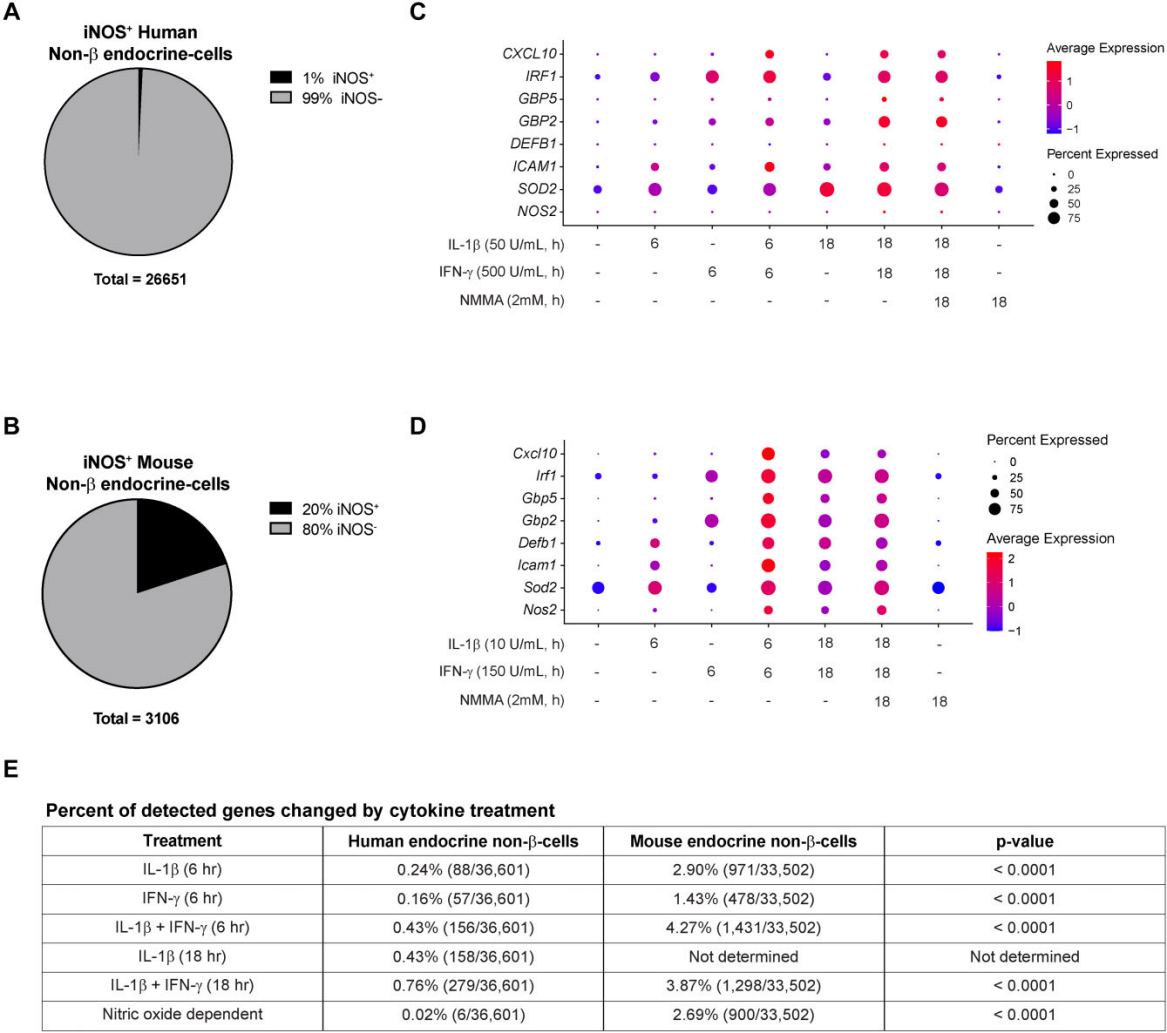
Cytokine responsiveness is also blunted in islet endocrine non-β-cells. (A and B) Pie chart showing the percentage of human (A) or mouse (B) endocrine non-β-cells from all samples that express iNOS mRNA. (C and D) Dot plots depicting the expression levels of and percentage of human (C) or mouse (D) endocrine non-β-cells expressing selected genes in response to each of the cytokine treatments. (E) Table summarizing the percentage of detected genes that were significantly changed by each cytokine treatment or genes that were significantly changed in a nitric oxide-dependent manner in human and mouse endocrine non-β-cells. *P*-value determined by Fisher’s exact test. Mouse data are from our previously published studies.^14^,^15^

We demonstrated that, in the mouse, endocrine non-β-cells (α-, δ-, and PP-cells) respond to cytokine stimulation in a manner that is nearly identical to β-cells.^14^,^15^ To test the hypothesis that human endocrine non-β-cells also respond to cytokines, we first determined the percentage of this population that expresses *NOS2* mRNA as a metric for “cytokine responsiveness.” To do this, we first isolated clusters 0, 2, 4, 5, 10, 11, and 13. Similar to our human β-cell population, only 1% (220/26,651) of the entire endocrine non-β-cell population expressed *NOS2* (Figure 3A). This is in stark contrast to our mouse studies, in which 20% (619/2,487) of the endocrine non-β-cells expressed *Nos2* (Figure 3B). Also like the human β-cells, the blunted response of the human endocrine non-β-cells extended beyond *NOS2* expression, with other expected cytokine-stimulated genes being blunted when compared to expression in mouse non-β endocrine cells (Figure 3C and D). We again performed differential expression analysis to determine the number of genes significantly different in each cytokine-treated population of endocrine non-β-cells compared to the untreated population (Table S3). In total, 88 genes (0.24% of the detected genes) were significantly changed after a 6-h treatment with IL-1β, 57 (0.16%) with IFN-γ, and 156 (0.43%) with IL-1β + IFN-γ. Following an 18-h treatment, 158 (0.43%) were changed in response to IL-1β and 279 (0.76%) in response to IL-1β + IFN-γ (Figure 3E). Six genes (0.02%) were changed in a nitric oxide-dependent manner (Figure 3E). These percentages are significantly lower than the expected values from mouse endocrine non-β-cells (Figure 3E), suggesting that all human islet endocrine cells, not just β-cells, have a reduced cytokine response compared to mouse islet endocrine cells.

### *NOS2* ^+^ Islet Endocrine Cells Are Enriched for Other Cytokine-Stimulated Genes

**Figure 4.**
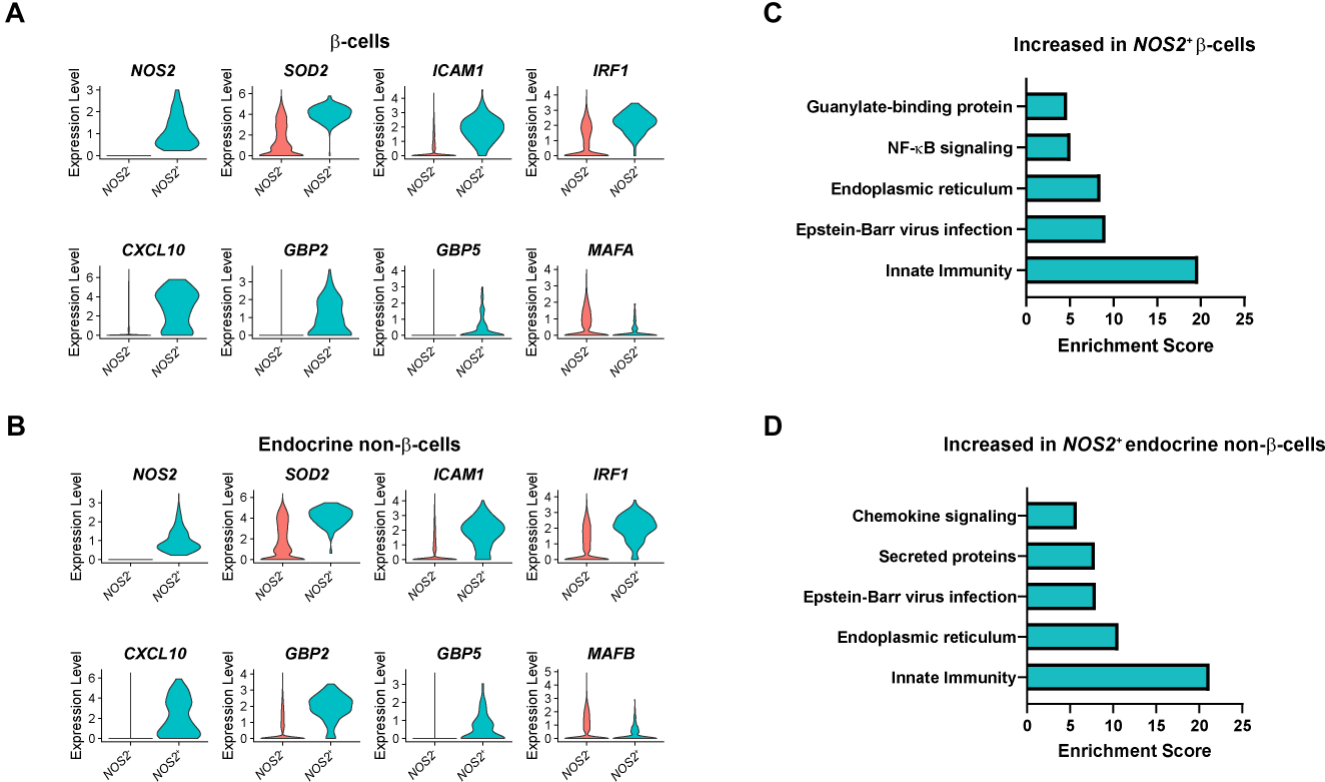
*NOS2* ^+^ islet endocrine cells are enriched for other cytokine-stimulated genes. (A and B) Violin plots showing the expression level of selected genes in human β-cells (A) or endocrine non-β-cells (B) expressing *NOS2* compared to those not expressing *NOS2*. All genes shown have *P*-values < 1 × 10^−8^. (C and D) Enriched categories of genes increased in human β-cells (C) or endocrine non-β-cells (D) expressing *NOS2* compared to those not expressing *NOS2*.

To test our original hypothesis that cytokines stimulate protective genes in human islet endocrine cells, we computationally isolated the β-cells expressing *NOS2* at any level (ie, > 0 reads of *NOS2* sequenced) from those not expressing *NOS2* or the endocrine non-β-cells expressing *NOS2* from those not expressing *NOS2* and performed differential expression analyses. Cells from all samples were used for this comparison. A total of 1,307 genes (1,219 enriched in the *NOS2*^+^ cells, and 88 enriched in the *NOS2*^−^ cells) were significantly different between the 2 β-cell populations, and 1,822 genes (1,620 enriched in the *NOS2*^+^ cells, and 202 enriched in the *NOS2*^−^ cells) were significantly different between the 2 endocrine non-β-cell populations (Tables S4 and S5). We found that the IL-1β-regulated genes *SOD2* and *ICAM1* and the IFN-stimulated genes *IRF1* and *CXCL10* had significantly higher expression levels in endocrine cells expressing *NOS2* compared to those not expressing *NOS2* (Figure 4A and B). Importantly, genes encoding antiviral guanylate-binding proteins *GBP2* and *GBP5*, which are IL-1β-stimulated in the mouse^14^, were also expressed at higher levels in the *NOS2*^+^ population (Figure 4A and B). Further, the identity genes *MAFA* and *MAFB*, which are known to be repressed by IL-1β in mouse islet endocrine cells,^14^ were significantly lower in human islet endocrine cells expressing *NOS2* compared to those not expressing *NOS2* (Figure 4A and B). Functional annotation clustering analysis demonstrated that genes falling into categories associated with cytokine signaling, including “Innate Immunity,” “Epstein-Barr virus infection,” “NF-κB signaling,” “guanylate-binding protein,” and “Chemokine signaling” were enriched in the *NOS2*^+^ human islet endocrine cells compared to the *NOS2*^−^ cells (Figure 4C and D). Together, these results demonstrate that although cytokine responses are generally blunted in human islet endocrine cells, cells that are responsive to cytokines (*NOS2*^+^ cells) have similar gene expression changes to those previously observed in the mouse, including stimulation of protective antiviral genes and repression of identity genes.

### Genes Encoding Ribosomal Proteins Are Enriched in *NOS2*^−^ β-Cells

**Figure 5.**
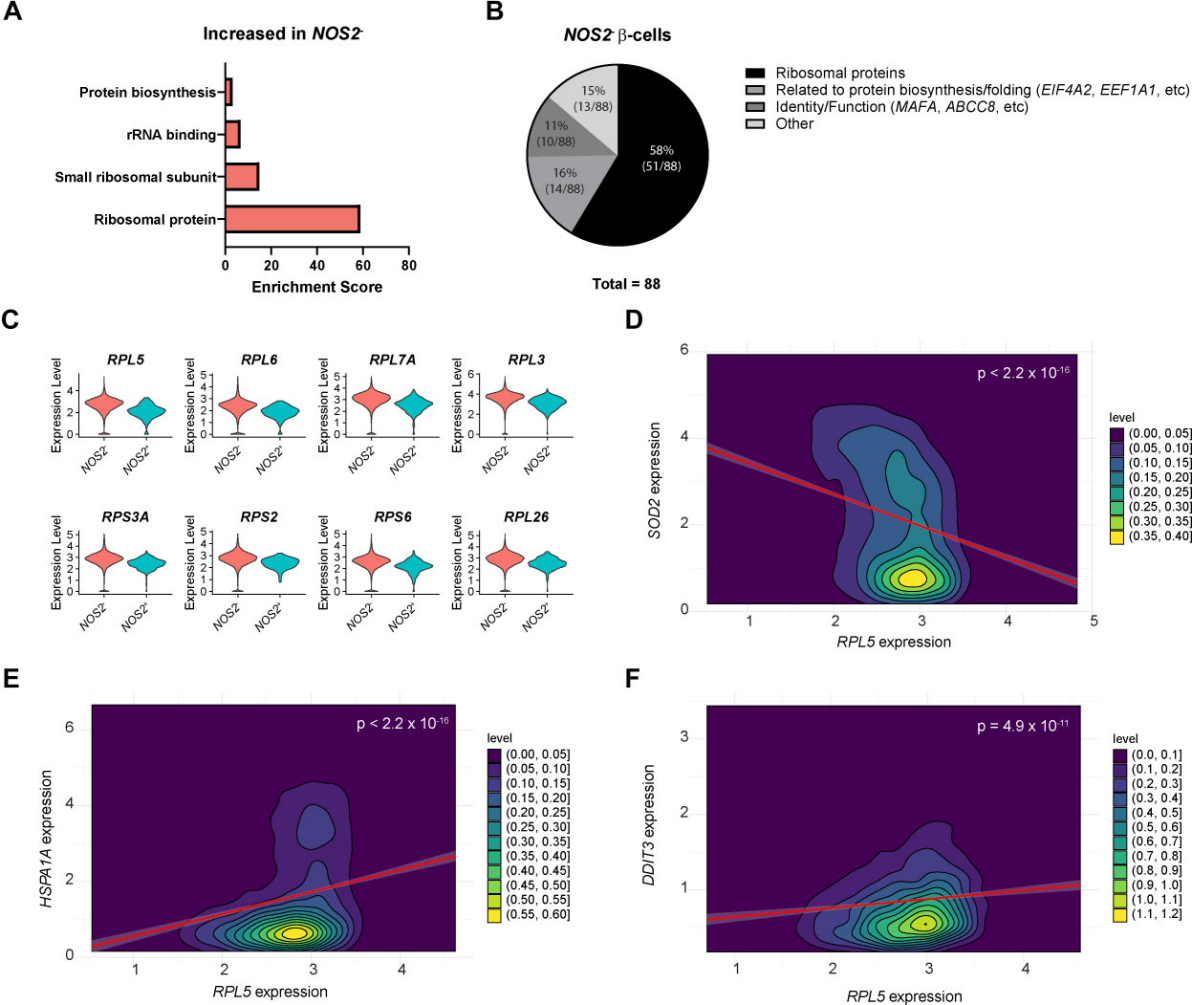
Genes encoding ribosomal proteins are enriched in *NOS2*^−^ β-cells. (A) Enriched categories of genes increased in human β-cells not expressing *NOS2* compared to those that are expressing *NOS2*. (B) Pie chart showing percentage of genes increased in *NOS2*^−^ β-cells that fall into selected categories. (C) Violin plots showing the expression level of selected ribosomal proteins in human β-cells expressing *NOS2* compared to those not expressing *NOS2*. All genes shown have *P*-values < 1 × 10^−5^. (D–F) Linear regression analyses showing the relationship between expression of *RPL5* and *SOD2* (*R*^2^ = 0.07931) (D), *HSPA1A* (*R*^2^ = 0.04593) (E), and *DDIT3* (*R*^2^ = 0.01323) (F) in human β-cells.

As shown in Figure 2A, less than 2% of the total β-cell population expresses detectable levels of *NOS2* mRNA, leading us to hypothesize that the remaining β-cells may have a common characteristic preventing them from responding to cytokine stimulation in the expected manner. To test this hypothesis, we again performed functional annotation clustering analysis, this time focusing on genes increased in β-cells not expressing *NOS2* as compared to β-cells expressing *NOS2*. Strikingly, the most enriched genes were those that encode ribosomal proteins (Figure 5A). In fact, of the 88 genes significantly higher in *NOS2*^−^ β-cells compared to *NOS2*^+^ β-cells, 58% encode ribosomal proteins and another 16% encode proteins that play other roles in protein biosynthesis or protein folding (Figure 5B). The ribosomal protein genes that are most enriched in the *NOS2*^−^ β-cell population are shown in Figure 5C, with ribosomal protein L5 (*RPL5*) being the gene in this category that is the most different compared to *NOS2*^+^ β-cells. *RPL5* is not increased by cytokine exposure, suggesting that it is basally high in *NOS2*^−^ β-cells (Figure S2 and Table S2). Linear regression analysis of the β-cells demonstrated a significant negative correlation between expression of *RPL5* and the IL-1β-stimulated gene *SOD2* (superoxide dismutase 2) (Figure 5D). Importantly, further analyses demonstrate that expression of *RPL5* is positively correlated with genes associated with cellular stress: *HSPA1A* (encodes the alpha subunit of heat shock protein 70) and *DDIT3* (encodes DNA damage inducible transcript 3, also known as CHOP) (Figure 5E and F). The negative association between *RPL5* and/or *HSPA1A* expression and cytokine responsiveness is mirrored by our original clustering analysis (Figure 1C) in that β-cells in clusters 7 and 12, characterized by enrichment for ribosomal proteins and heat shock proteins, respectively, have lower expression of cytokine-stimulated genes *SOD2, ICAM1*, and *IRF1* (Figure S3 and Table S1). These data together suggest that high expression of ribosomal proteins is an indicator of cellular stress and is negatively correlated with cytokine response in β-cells.

### High Expression of *RPL5* Predicts Failure to Respond to Cytokines in Human β-Cells

**Figure 6.**
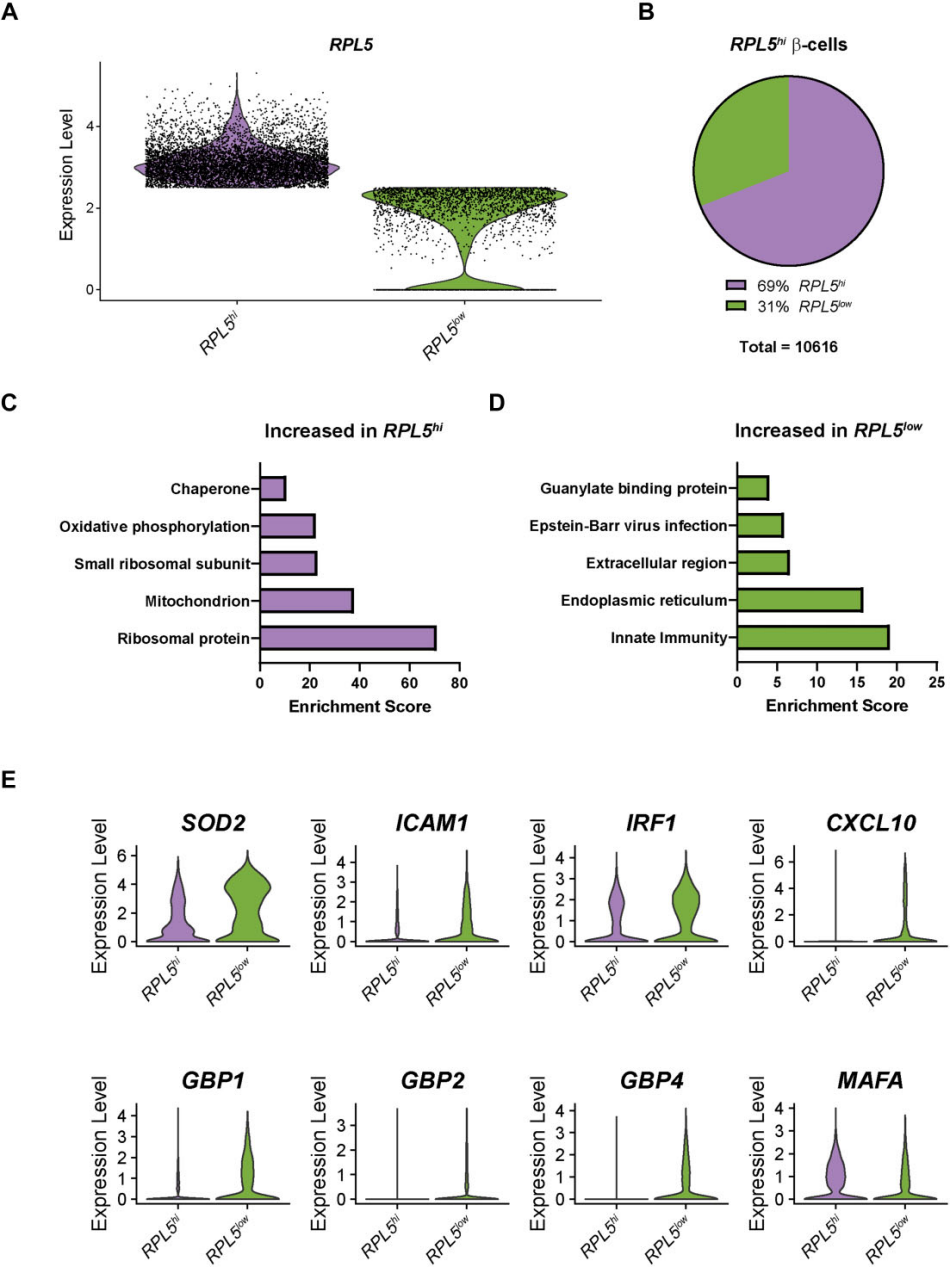
High expression of *RPL5* predicts failure to respond to cytokines in human β-cells. (A) Violin plot showing expression level of *RPL5* in human β-cells with “high” *RPL5* expression (*RPL5^hi^*) compared to those with “low” *RPL5* expression (*RPL5^low^*). (B) Pie chart showing the percentage of *RPL5^hi^* and *RPL5^low^* β-cells. (C and D) Enriched categories of genes increased in *RPL5^hi^* β-cells compared to *RPL5^low^* β-cells (C) and vice versa (D). (E) Violin plots showing the expression level of selected genes in *RPL5^hi^* β-cells compared to *RPL5^low^* β-cells. All genes shown have *P*-values < 1 × 10^−36^.

Since comparison of *NOS2*-expressing β-cells to *NOS2*-non-expressing β-cells revealed a correlation to *RPL5* expression (Figure 5), we hypothesized that β-cells with high expression of *RPL5* would be less likely to respond to cytokines than those with low expression of *RPL5*. To test this hypothesis, we computationally separated the β-cells from all samples into “*RPL5^hi^*” and “*RPL5^low^*” populations, using an expression level of 2.5 as our cutoff (Figure 6A). Over two-thirds of the β-cells fell into the “*RPL5^hi^*” category, with the other third falling into the “*RPL5^low^*” category (Figure 6B). We performed differential expression analysis and functional annotation clustering analysis to determine which categories of genes are different between the 2 β-cell populations. A total of 2,840 genes were significantly different between the *RPL5^hi^* and *RPL5^low^* β-cells (Table S6). As expected, gene categories of “ribosomal protein,” “small ribosomal subunit,” and “chaperone” were enriched in the *RPL5^hi^* β-cells (Figure 6C). Interestingly, categories of “innate immunity,” “Epstein-Barr virus infection,” and “guanylate binding protein” were enriched in the *RPL5^low^* β-cells (Figure 6D). Consistent with this, IL-1β-stimulated genes *SOD2* and *ICAM1* and IFN-stimulated genes *IRF1* and *CXCL10* were significantly higher in the *RPL5^low^* β-cells (Figure 6E). Importantly, antiviral guanylate-binding proteins *GBP1, GBP2*, and *GBP4* were also increased in the *RPL5^low^* β-cells, while identity gene *MAFA* was increased in the *RPL5^hi^* β-cells (Figure 6E). These results suggest that low expression of *RPL5* predicts cytokine responsiveness, and β-cells with low expression of this ribosomal protein are more likely to stimulate protective gene expression in response to cytokine exposure.

### High Expression of *RPL5* Predicts Failure to Respond to Cytokines in Human Endocrine Non-β-Cells

**Figure 7.**
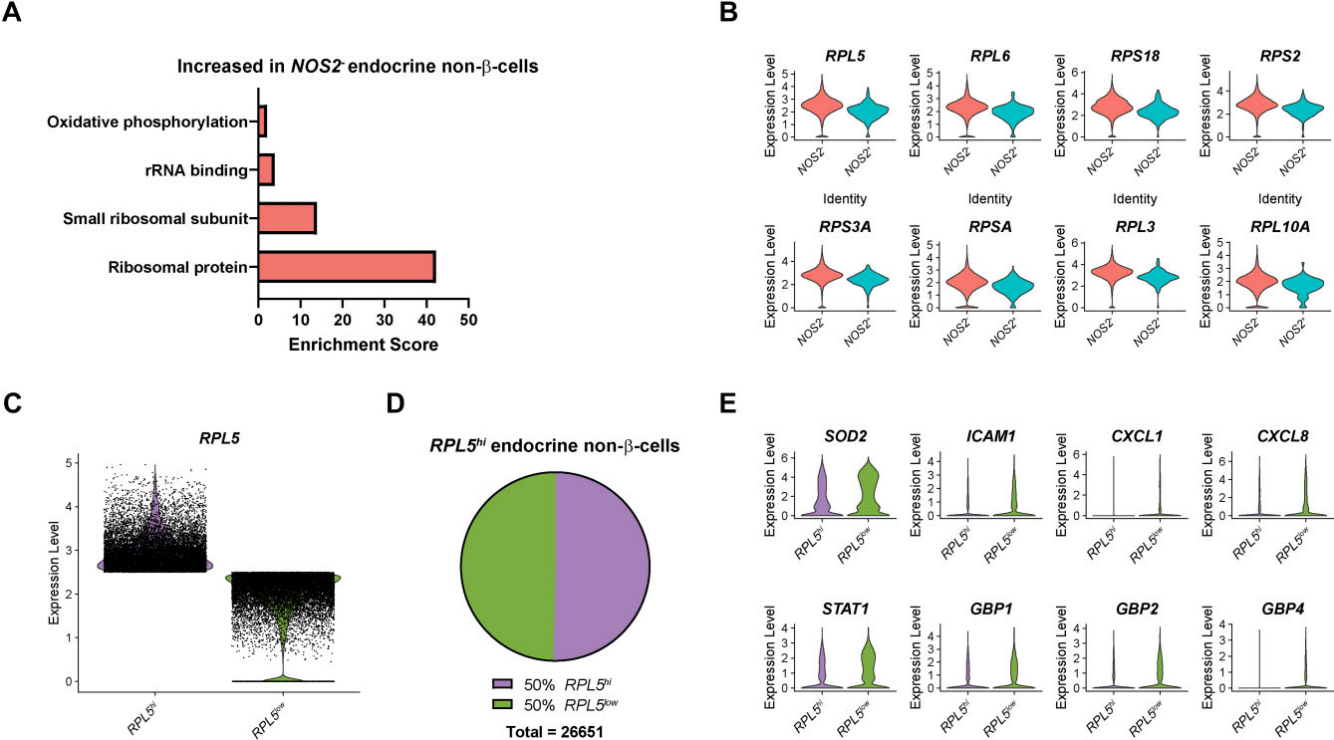
High expression of *RPL5* predicts failure to respond to cytokines in endocrine non-β-cells. (A) Enriched categories of genes increased in human endocrine non-β-cells not expressing *NOS2* compared to those that are expressing *NOS2*. (B) Violin plots showing the expression level of selected ribosomal proteins in human endocrine non-β-cells expressing *NOS2* compared to those not expressing *NOS2*. All genes shown have *P*-values < 1 × 10^−8^. (C) Violin plot showing expression level of *RPL5* in human endocrine non-β-cells with “high” *RPL5* expression (*RPL5^hi^*) compared to those with “low” *RPL5* expression (*RPL5^low^*). All genes shown have *P*-values < 1 × 10^−86^. (D) Pie chart showing the percentage of *RPL5^hi^* and *RPL5^low^* endocrine non-β-cells. (E) Violin plots showing the expression level of selected genes in *RPL5^hi^* endocrine non-β-cells compared to *RPL5^low^* endocrine non-β-cells.

Like in the β-cells, genes that are enriched in endocrine non-β-cells that are not expressing *NOS2* largely fall into the category of ribosomal proteins and other categories related to protein biosynthesis (Figure 7A). Since *RPL5* is the gene of this category that is most different between the *NOS2*-expressing and non-expressing populations (Figure 7B), we hypothesized that its expression may be able to predict cytokine responsiveness in the endocrine non-β-cells like it did in the β-cells. To test this hypothesis, we computationally separated the endocrine non-β-cells from all samples into “*RPL5^hi^*” and “*RPL5^low^*” populations, using an expression level of 2.5 as our cutoff (Figure 7C). In contrast to the β-cells, the endocrine non-β-cells were split evenly into the “*RPL5^hi^*” and “*RPL5^low^*” categories (Figure 7D). A total of 8,786 genes were significantly different between the *RPL5^hi^* and *RPL5^low^* endocrine non-β-cells (Table S7). Among those that were higher in the *RPL5^low^* population were IL-1β-stimulated genes *SOD2* and *ICAM1*, IFN-stimulated genes *CXCL1, CXCL8*, and *STAT1*, and antiviral guanylate-binding proteins *GBP1, GBP2*, and *GBP4* (Figure 7E). This observation indicates that high expression of ribosomal proteins is negatively correlated with cytokine responsiveness not only in human β-cells but also in endocrine non-β-cells and that low expression of *RPL5* predicts a higher likelihood of responding to cytokine stimulation in all islet endocrine cell types.

### High Expression of *RPL5* Predicts Failure to Respond to Cytokines in Human Non-Endocrine Cells

**Figure 8.**
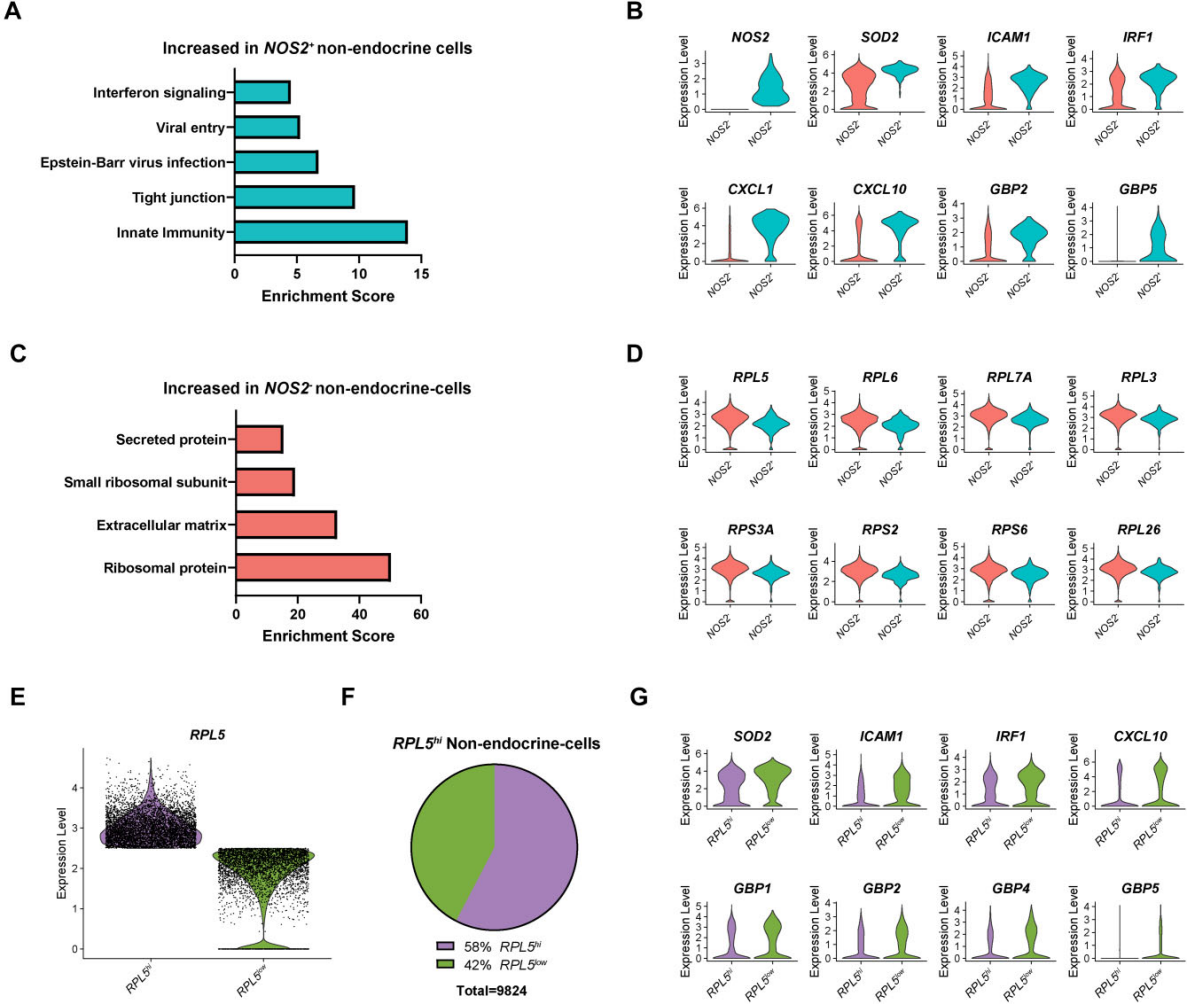
High expression of *RPL5* predicts failure to respond to cytokines in non-endocrine cells. (A and C) Enriched categories of genes increased in human non-endocrine cells expressing *NOS2* compared to those non expressing *NOS2* (A) or vice versa (C). (B and D) Violin plots showing the expression level of selected genes in human non-endocrine cells expressing *NOS2* compared to those not expressing *NOS2*. (E) Violin plot showing expression level of *RPL5* in human non-endocrine cells with “high” *RPL5* expression (*RPL5^hi^*) compared to those with “low” *RPL5* expression (*RPL5^low^*). (F) Pie chart showing the percentage of *RPL5^hi^* and *RPL5^low^* non-endocrine cells. (G) Violin plots showing the expression level of selected genes in *RPL5^hi^* non-endocrine cells compared to *RPL5^low^* non-endocrine cells.

Approximately 20% of our dataset was made up of non-endocrine cells (Figure 1C). Of these, almost 4% (360 out of 9,824) expressed *NOS2* (data not shown). When we computationally separated the non-endocrine cells from all samples into *NOS2*^+^ and *NOS2*^−^ populations and performed differential expression analysis, 3,227 genes were found to be significantly different between the 2 groups (Table S8). Functional annotation clustering analysis revealed that, like in the endocrine cell populations, categories of “innate immunity,” “Epstein-Barr virus infection,” “viral entry,” and “interferon signaling” were increased in the *NOS2*^+^ non-endocrine cells (Figure 8A). Among the genes increased in this population were IL-1β-stimulated genes *SOD2* and *ICAM1*, IFN-stimulated genes *IRF1, CXCL1*, and *CXCL10*, and antiviral guanylate-binding proteins *GBP2* and *GBP5* (Figure 8B). Conversely, categories of “ribosomal protein” and “small ribosomal subunit” were increased in the *NOS2*^−^ non-endocrine cells (Figure 8C), also consistent with our observations in the endocrine cells. While *RPL5* was not the most differentially expressed ribosomal protein between the 2 non-endocrine cell populations, it was still among those of this category that were different (Figure 8D). To test the hypothesis that this gene might predict cytokine responsiveness in the non-endocrine cells of the islet, we divided this population into “*RPL5^hi^*” and “*RPL5^low^*” subsets, using an expression level of 2.5 as our cutoff (Figure 8E). Nearly 60% of the non-endocrine cells fell into the “*RPL5^hi^*” category, while the remaining 40% fell into the “*RPL5^low^*” category (Figure 8F). Differential expression analysis identified 5,391 genes that were significantly different between the 2 populations (Table S9). Among those that were higher in the *RPL5^low^* population were IL-1β-stimulated genes *SOD2* and *ICAM1*, IFN-stimulated genes *IRF1* and *CXCL10*, and antiviral guanylate-binding proteins *GBP1, GBP2, GBP4*, and *GBP5* (Figure 8B). Together, our analysis of the non-endocrine cells of our dataset demonstrates that high expression of genes encoding ribosomal proteins is negatively associated with cytokine responsiveness in all islet cell types.

### Donor Cellular Stress and Cytokine Responsiveness

**Figure 9.**
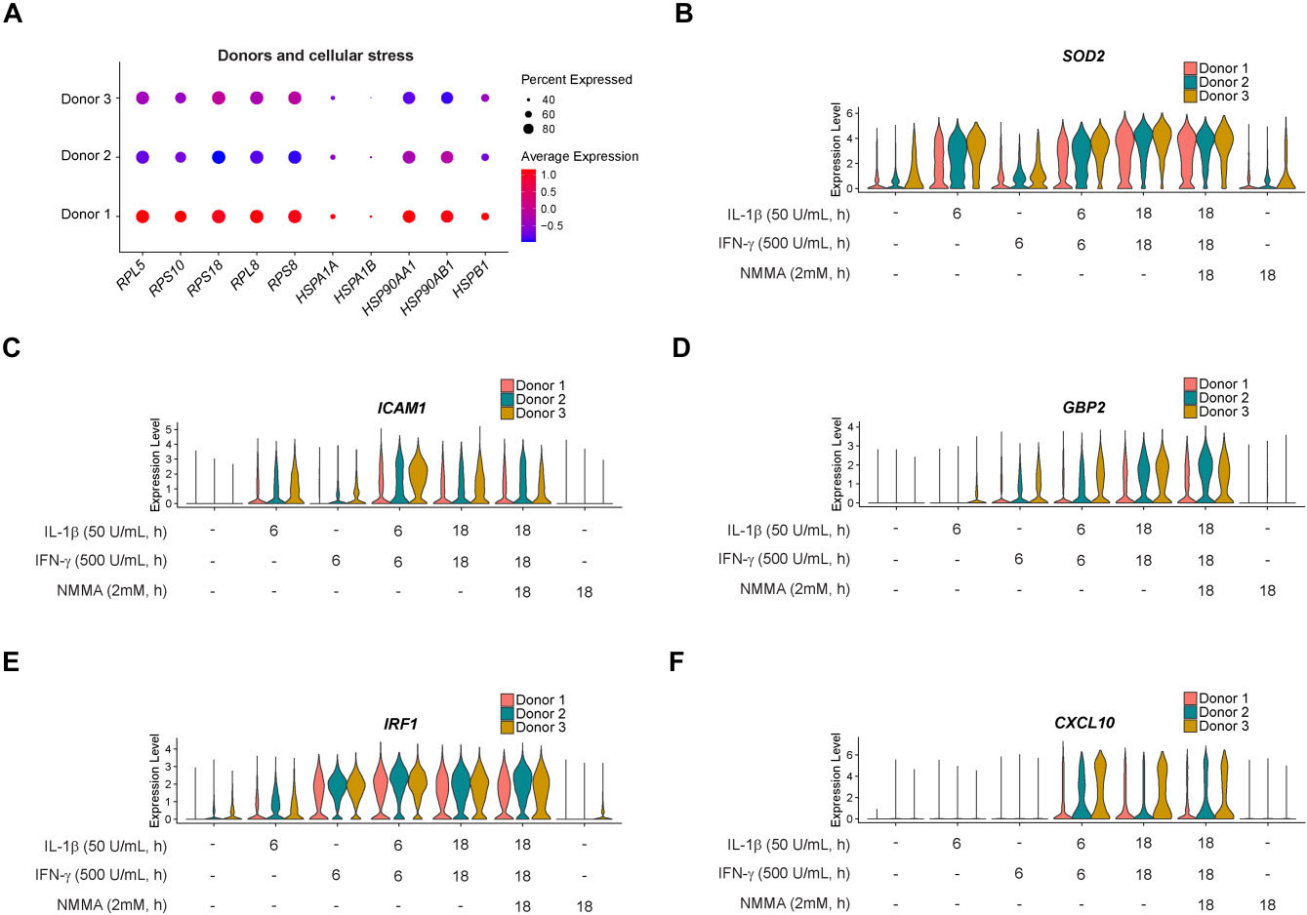
Donor cellular stress levels and cytokine responsiveness. (A) Dot plot showing the expression levels of and percentage of cells from each of the 3 donors expressing selected ribosomal proteins and heat shock proteins. (B–F) Split violin plots showing the expression of *SOD2* (B), *ICAM1* (C), *GBP2* (D), *IRF1* (E), and *CXCL10* (F) in response to cytokine stimulation in human islets from each of the 3 donors. All genes shown have *P*-values < 1 × 10^−15^.

To determine potential differences in cytokine responsiveness among our 3 donors, we first determined markers that were enriched in each of our 3 samples using all cells captured (Table S10). Genes encoding ribosomal proteins, including *RPL5, RPS10, RPS18, RPL8*, and *RPS8*, as well as genes encoding heat shock proteins, like *HSPA1A, HSPA1B, HSP90AA1, HSP90AB1*, and *HSPB1*, were significantly higher in cells from Donor 1 compared to cells from the other 2 donors (Figure 9A). Importantly, cells from Donor 1 were less responsive to cytokines compared to the cells from the other 2 donors. This was evidenced by reduced expression of IL-1β-stimulated genes *SOD2* and *ICAM1*, antiviral guanylate binding protein *GBP2*, and IFN-stimulated genes *IRF1* and *CXCL10* (Figure 9B–F). Interestingly, Donor 1 died from head trauma while the other 2 donors died from either a stroke or an anoxic event (Figure 1A). While our sample size is too small to make robust conclusions, these observations suggest that differences in donor characteristics, such as cause of death, may underly differences in cellular stress and islet cytokine responsiveness.

## Discussion

The inflammatory cytokine IL-1β is primarily thought to damage pancreatic β-cells due to observations that IL-1β and IL-1β-derived nitric oxide inhibit mitochondrial oxidation and glucose-stimulated insulin secretion and cause DNA damage.^8–13^ However, we have recently shown that there are physiological roles for IL-1β and nitric oxide signaling in β-cells that include the stimulation of antiviral and protective genes by IL-1β and inhibition of viral replication by nitric oxide.^14–17^ Because these studies were performed using mouse islets and because differences in the responses of mouse and human islets to IL-1β have been reported,^23–25^,^30^,^31^ here, we aimed to test the hypothesis that IL-1β stimulates protective genes in human pancreatic islet endocrine cells.

We found that gene expression changes in both β-cells and endocrine non-β-cells following cytokine exposure were markedly blunted in the human islets compared to our previous observations in mouse islets (Figures 2 and 3).^14^,^15^ This was evident not only by a smaller percentage of cells expressing *NOS2* but also by a smaller percentage of differentially expressed genes. However, when we computationally isolated the β-cells and the endocrine non-β-cells with detectable *NOS2* expression and compared them to cells without detectable *NOS2* expression, we observed significantly higher expression of known cytokine-stimulated genes, including IL-1β-stimulated antiviral guanylate-binding proteins *GBP2* and *GBP5* (Figure 4). This critical observation suggests that, although the global response to cytokine stimulation is blunted in the human islet endocrine cells assayed here, a small percentage of them respond to IL-1β by increasing protective gene expression. It also suggests that there may be a common feature of the *NOS2*^−^ cells preventing them from responding to cytokines. The identification of these 2 populations of cells (*NOS2*-expressing and *NOS2* non-expressing) was only possible because of our single-cell approach. It is also worth noting that our use of the minimum cytokine concentrations necessary to stimulate nitric oxide production in human islets may have led us to underestimate the size of the *NOS2*^+^ population.^12^,^40^ In other words, “cytokine responsiveness” may have increased if we had used higher concentrations of the cytokines. However, the concentrations used here are still greater than those used in our previous mouse islet studies, indicating that, even at higher cytokine concentrations, human islets are still less responsive to cytokines than mouse islets.^14^,^15^

By comparing *NOS2*^+^ and *NOS2*^−^ populations of β-cells (Figure 5), endocrine non-β-cells (Figure 7), and non-endocrine cells (Figure 8), we identified a common association among all *NOS2*^−^ populations: higher expression of genes encoding ribosomal proteins than the respective *NOS2*^+^ population. This difference was particularly striking in β-cells where nearly 60% of the genes that were significantly enriched in the *NOS2*^−^ population encoded ribosomal proteins (Figure 5B). We further demonstrated that expression of ribosomal protein L5 (*RPL5*) is negatively correlated with expression of an IL-1β-regulated gene, *SOD2*, and is positively correlated with expression of “stress” genes heat shock protein 70 (*HSPA1A*) and CHOP (*DDIT3*) (Figure 5D–F). Most importantly, high expression of *RPL5* predicted failure to respond to cytokine stimulation in β-cells (Figure 6), endocrine non-β-cells (Figure 7), and non-endocrine cells (Figure 8), suggesting that *RPL5* may provide a novel marker of “cellular stress” in human islets.

This association between cellular stress and blunted cytokine signaling is consistent with previous observations by us and others that induction of heat shock stress or ER stress prevents iNOS expression following cytokine stimulation in rodent and human islets.^30^,^33–35^ In our previous scRNA-seq studies using mouse islets, we observed populations of β-cells characterized by high expression of heat shock and ribosomal proteins that failed to respond to IL-1β and IFN-γ exposure by increasing *Nos2* mRNA, further emphasizing this negative relationship between cellular stress and cytokine signaling.^14^,^15^ It is critical to emphasize that cells in the current study with high expression of *RPL5* had lower expression not only of IL-1β-stimulated genes (like *SOD2* and *ICAM1*) but also had blunted expression of IFN-stimulated genes (like *IRF1, CXCL10*, and *STAT1*). This observation demonstrates that the negative effects of cellular stress on cytokine signaling are not limited to IL-1β but apply to cytokine responses more broadly, consistent with previous studies.^34^,^35^

We are not the first to report evidence of high levels of cellular stress in human islet preparations.^30^,^31^,^41–43^ However, the cause of this stress remains unclear. Some have suggested that the stress may originate during the cold storage time following organ explantation or during the process of islet isolation.^43–45^ Others have suggested that islets exhibit cellular stress even before the islet isolation process.^46^ Here, we observed differences in cytokine responsiveness among our 3 islet samples that were correlated with differences in expression levels of genes encoding ribosomal proteins and heat shock proteins (Figure 9A). Interestingly, islets from the donor who died from head trauma (Donor 1) had higher levels of “stress-associated” genes and had a blunted cytokine response compared to islets from the donors who died from a stroke or an anoxic event (Figure 9B–F). This observation is consistent with the documented induction of a “cytokine storm” following traumatic brain injury in patients.^47^,^48^ This systemic cytokine release likely leads to local nitric oxide production in the islet microenvironment, which is known not only to stimulate expression of heat shock proteins, but also to blunt subsequent cytokine signaling in the β-cell.^33^,^34^ While our sample size here is too small to make any definitive conclusions, we would like to suggest that donor cause of death may contribute to cellular stress levels and cytokine responsiveness of human islet preparations. However, we cannot exclude the possibility that factors other than donor cause of death contributed to differences in stress levels observed among our 3 islet samples. Additional factors include different genetic and phenotypic makeup, disease profiles, usage of pharmaceuticals of donors, as well potential differences in variables associated with islet isolation, from cold storage time of donor pancreas to days in culture. Finally, it is possible that human islets intrinsically express higher levels of stress-response genes than rodent islets. Indeed, Welsh *et al*. observed that human islets express higher levels of HSP70 than rat islets even following 4 wk of transplantation.^31^

Regardless of the cause of the stress that is so common to cadaveric human islets, we postulate that these high levels of stress may explain reported differences between the responses of human and rodent β-cells to cytokines.^23–25^ The observation presented here that β-cells with lower expression of a stress-associated gene (*RPL5*) respond to cytokines in the same manner as rodent β-cells (by increasing expression of antiviral guanylate-binding proteins) strongly suggests that cellular stress characteristic of many human islet preparations has prevented a complete understanding of the cytokine responses of human β-cells. As human islets are increasingly being used in cytokine studies, our findings indicate that it is imperative to assess expression of “stress” genes, like *RPL5* and *HSPA1A*, before making conclusions regarding the effects, or lack of effects, of cytokines on human β-cell viability and function.

## Supplementary Material

zqae015_Supplemental_Files

## Data Availability

Sequencing data from this publication have been deposited in NCBI GEO database under accession number GSE251730.
