# Supplementary material for: Single Cell RNAseq Analysis of Cytokine-Treated Human Islets: Association of Cellular Stress with Impaired Cytokine Responsiveness
Source: Function (Oxf). 2024 Mar 27;5(4):zqae015. doi: 10.1093/function/zqae015 (PMC11237896; doi:10.1093/function/zqae015)
Supplement: zqae015_Supplemental_Files [file zqae015_supplemental_files.zip › Supplemental_Figures.docx]

**
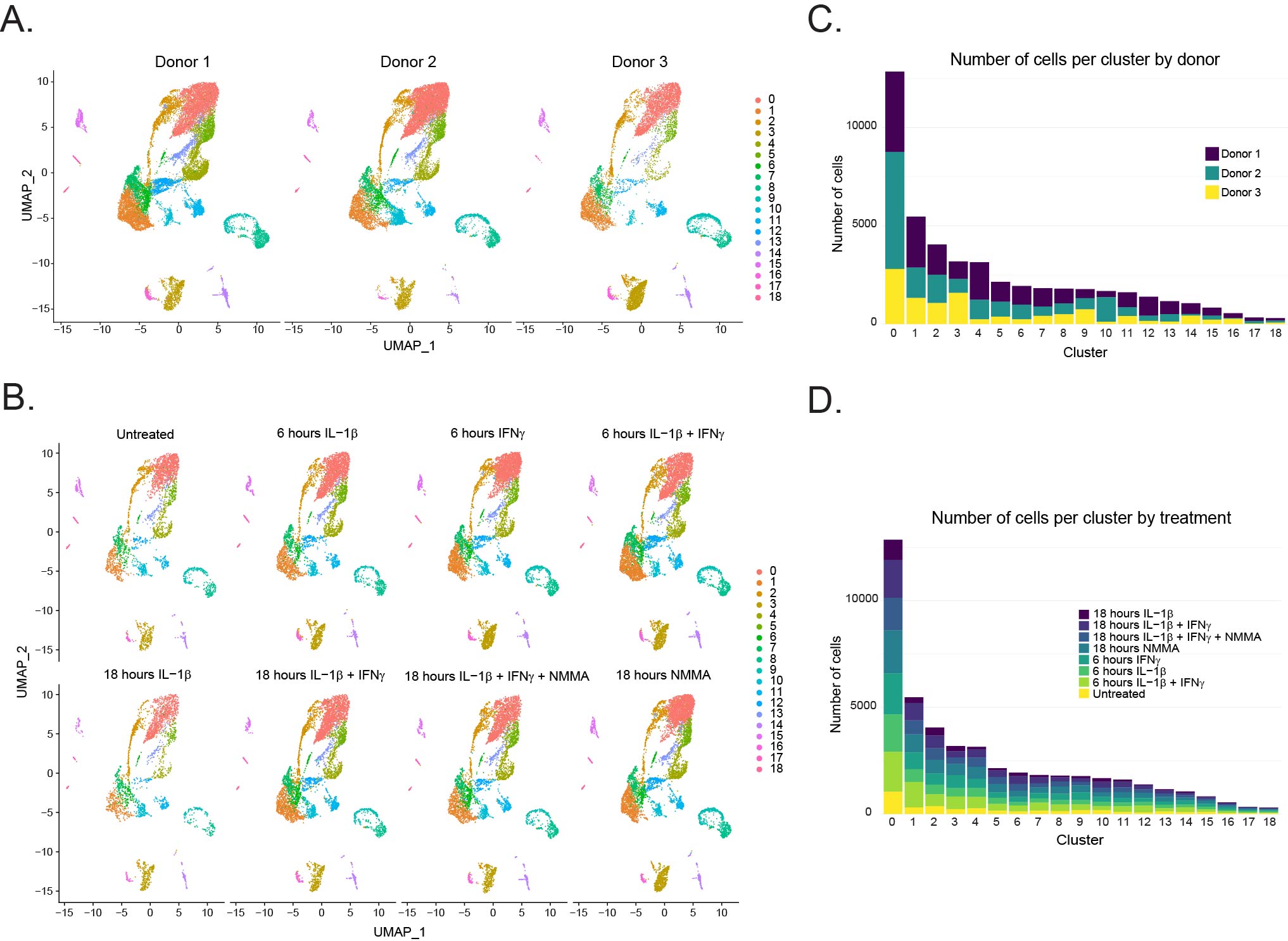
**

**Figure S1: Distribution of clusters by donor and by treatment. (A-B)** Split UMAP plots showing the contribution of cells to each cluster based on donor of origin (A) or by experimental treatment (B). Cluster identity is shown to the right of the plot. **(C-D)** Stacked bar plots showing the number of cells from each donor (C) or from each experimental treatment group (D) in each cluster.

**
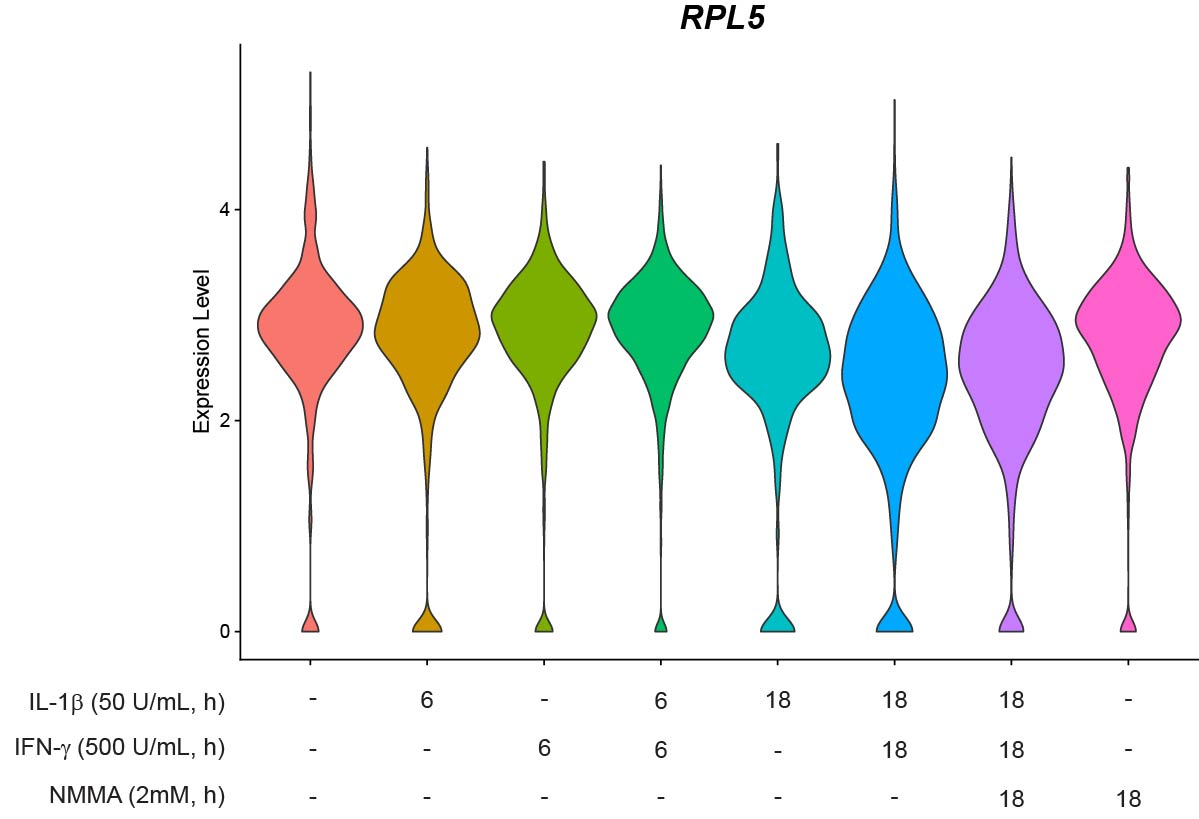
**

**Figure S2: *RPL5* expression in *NOS2^-^* β-cells.** Violin plot showing the expression of *RPL5* in β-cells not expressing *NOS2* (*NOS2^-^*) in response to each experimental treatment.

**
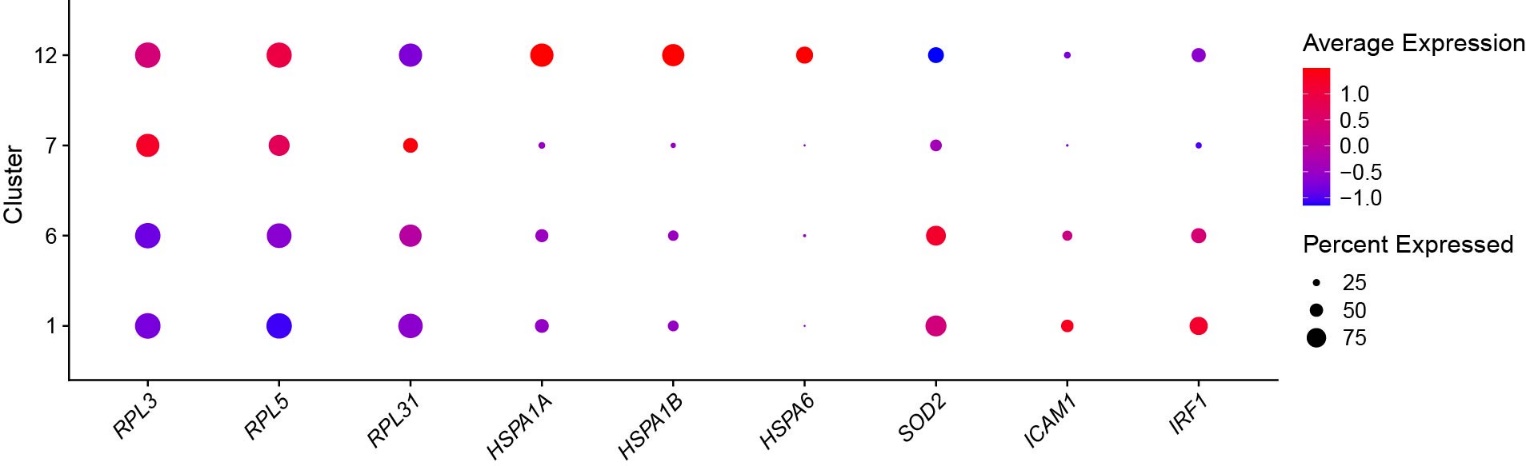
**

**Figure S3: Genes of interest in β-cell clusters.** Dot plot showing the expression levels of and percentage of cells from each of the four β-cell clusters expressing selected ribosomal proteins, heat shock proteins, and cytokine-stimulated genes.
